# SuperVive-Comunidad App: Advancing Integrative Health Equity for the Hispanic Community Through Social Connection

**DOI:** 10.1177/27536130251345428

**Published:** 2025-05-23

**Authors:** Arlene Betancourt

**Affiliations:** 1 Department of Physician Assistant Studies, 171780 UT Southwestern School of Health Professions, Dallas, TX, USA; 2 Department of Internal Medicine, UT Southwestern Medical Center, Dallas, TX, USA

**Keywords:** community health partnerships, integrative health equity, health outcomes, health disparities, digital health, language concordance

## Abstract

Barriers to the use of integrative medicine modalities are complex, with associated disparities in awareness, access, and utilization within marginalized communities. This article details our experience in creating a virtual community designed to empower Hispanic women in the US to lead healthier lives through culturally aware health education in Spanish. Our digital health programs utilize a mind-body medicine approach to foster positive social connections and promote integrative health equity within the Hispanic community living in the US.

## Introduction

The Hispanic population in the US has seen exponential growth over the past decades and is now the largest ethnic minority in the US, accounting for 19% of the total population.^1^ Hispanic people in the US are primarily employed in service industries including construction, agriculture and hospitality,^2^ and have the highest prevalence of workers without sick time benefits, with only 56.6% having access to paid sick leave.^3^ According to the Pew Research Center, 18% of the US Hispanic population lived in poverty in 2021.^4^ In 2023, Hispanic people in the US had the second highest uninsured rates, with 17.9% percent lacking private or public health insurance among individuals ages 65 and below.^5^

Educational achievement and the ability to speak English proficiently are higher for US-born individuals compared to immigrants of Hispanic origin.^4^ Seventy percent of the Hispanic population in the US speak a language other than English at home, most commonly Spanish.^1^ Limited English proficiency in Hispanic patients and the limited availability of language-concordant clinicians have been recognized as contributing factors to delayed access to healthcare, mistrust, and poor health outcomes.^6^ With a predominately English-language health care system, Hispanic people with limited English proficiency face challenges while navigating care, including difficulty communicating, understanding and participating in care, leading to delays in seeking health care and lower rates of preventative screenings when compared to the non-Hispanic White population.^7^

The benefits of language-concordant healthcare are well-documented. Studies have shown that when patients with limited English proficiency receive care from language-concordant physicians, the patients report better satisfaction, enhanced trust,^8^ and better process measures including improved blood pressure, HbA1C, and LDL levels.^6^ Patients with limited English proficiency who receive care from language-concordant physicians are more likely to receive education about their care and show better medication adherence.^6^ A systematic review of the literature suggests that language-concordant care can lead to better satisfaction and empowerment among patients, better therapeutic relationships between patient and clinician and positive health outcomes.^6^

Implementing language-concordant and culturally aware health care for Hispanic people with limited English proficiency (LEP) presents significant challenges in the US health care system. The Hispanic community is underrepresented in healthcare with less than 7% of physicians and 9% of other healthcare professionals identifying as Hispanic in the US.^9^ Additional challenges include limited health literacy among patients with LEP and high rates of underinsured or uninsured patients among the Hispanic people living in the US.^10^ A better integration between health, educational and community services has been proposed to advance health literacy and health outcomes for Hispanic people with limited English proficiency.^11^

The use of complementary medicine in the US increased from 19.2% in 2002 to 36.7% in 2022, with meditation, yoga and massage therapy showing the most significant increase in utilization.^12^ In addition, among participants reporting the use of one or more of the complementary approaches studied, the percentage reporting use for pain management increased from 42.3% in 2002 to 49.2% in 2022.^12^

In the National Health Interview Survey (NHIS) report for 2012, 22% of Hispanic people reported using complementary modalities vs 37.9% of non-Hispanic white people.^13^ Between 2002 and 2012, there was a trend toward less use of integrative modalities among marginalized communities and individuals with lower education and financial resources.^13^ The NHIS 2017 data showed that non-Hispanic white adults were more likely to use yoga, meditation, and chiropractors compared with Hispanic and non-Hispanic black adults.^14^

Barriers to the use of integrative medicine and complementary modalities are complex and multifaceted. Awareness, availability, accessibility, and affordability have been identified as potential barriers to integrative medicine use among marginalized communities.^15^ In a recent qualitative study, barriers including funding and resource allocation continue to hinder initiatives to reduce disparities in complementary and integrative health utilization within marginalized communities.^16^ The intentional design of community partnerships has been shown to positively impact the access and utilization of integrative and complementary modalities.^16^ Integrative community-based programs have shown significant potential to reduce health care disparities by addressing both medical and social determinants of health. These programs involve integrated care models focusing on the creation of multidisciplinary and cross-sector collaborations to improve health equity.^17^

Positive relationships and social connection are thought to promote beneficial biological, psychological and behavioral changes; by reducing stress and inflammation and contributing to purpose and meaning, which translate into positive health behaviors.^18^ The COVID pandemic highlighted the need for culturally relevant health education and community support.^19^ In the national advisory on the benefits of social connection in health and healing released in 2023, the US Surgeon General calls governments to invest in institutions that bring people together, support the development of pro-connection technologies, and expand the research and knowledge on social connection and healing.^20^

Integrative medicine group visits have emerged as a promising approach to enhance social support and improve access to integrative care and reduce health disparities.^21^ These group visits combine medical care with complementary therapies and social support, creating a whole person and community-centered model of care.^22,23^ During the COVID pandemic, Thompson-Lastad et al describe the implementation of integrative group medical visits in Federally Qualified Health Centers, which included food insecurity screening, produce prescriptions and nutrition education.^23^ While 57% of the enrolled participants were of Hispanic origin, only 29% of the Hispanic participants utilized both the Food Farmacy and the Integrative Group Medical Visits (IGMV) program likely due to limited Spanish-language IGMVs during this period.^23^

This article aims to detail the design and implementation of a digital health platform developed to increase access to culturally relevant integrative health education in Spanish while promoting a sense of community and empowerment among the Hispanic population in the United States.

## Methods

### Positionality

The author was born in Puerto Rico and self-identifies as a Hispanic woman. She completed residency training in internal medicine in Dallas, Texas, and is fellowship trained and board certified in integrative medicine. She has served on Rosa es Rojo’s board of directors since 2021 and contributed to the curricular design, implementation and facilitation of the SuperVive empowerment virtual class series from March 2023 to April 2024.

Rosa es Rojo was created to address the gap in health education and prevention resources specifically designed for the Hispanic community in the U.S. Founded by Aidee Granados following her personal experience as a Mexican immigrant living with breast cancer in a new country, the organization evolved from a blog to a registered nonprofit in 2016. Rosa es Rojo’s 2 signature programs - El Camino Rojo or The Rojo Way (TRW) and Supervive Comunidad app (SV) - promote behavioral change across 4 key domains: nutrition, mental health, physical activity, and empowered health. The nutrition, mental health and physical activity programs incorporate education on accessible integrative modalities to support a holistic approach to health and wellbeing. The Empowered Health pillar, a signature feature of the programs, aims to provide the participants with the tools and community support they need to actively participate in their health care journey. All content and experiences are designed and delivered in Spanish in their entirety, specifically tailored for Hispanic women ages 18 and above living in the US.

### The Rojo Way

TRW is a 23-hour program that utilizes experiential learning to break down complex health concepts into simple and actionable goals.

### TRW Recruitment

The participants learn about TRW from former graduates, social media, and/or community partners. TRW tuition cost is $735 per participant, funded entirely by donors and community partners. Since its establishment, most TRW donations have been restricted to self-identified Hispanic women ages 18 and above. In addition, a gift might be restricted to Hispanic women living in specific zip codes or counties. When recruiting in collaboration with community partners, the partnering organization champions the identification and recruitment of participants. TRW typically operates 8-10 virtual or in person cohorts annually, serving over 250 Hispanic women.

### TRW Program Training

TRW program encompasses 19 h of interactive group workshops followed by 4 h of individual mentoring and goal setting facilitated by a program ambassador. TRW graduates are invited to attend one of 2 biannual graduation ceremonies where individual and collective successes are celebrated.

Program delivery relies on a peer-education model utilizing experiential learning methodologies. Each cohort benefits from a primary facilitator and a co-facilitator or ambassador. TRW facilitators are former TRW graduates and program ambassadors who have obtained additional certification in health coaching. TRW graduates who are interested in becoming program ambassadors receive a minimum of 12 h of additional training in areas including goal setting, facilitation, and community outreach. The program ambassadors serve as co-facilitators for TRW workshops and provide 4 h of individual mentoring to the participants before graduation. To date, Rosa es Rojo has trained 35 TRW program ambassadors with 15 actively working with the program at the time of publication.

### SuperVive Comunidad App

Since its beginning, TRW has been supported by a podcast and YouTube video series. With the COVID pandemic, all Rosa es Rojo programs became virtual which facilitated reaching a larger audience and receiving support from community partners including United Way of Metropolitan Dallas, the Echoing Green Fellowship, and the Roddenberry Foundation. In 2022, SuperVive Comunidad app was launched as a pilot to empower the health and well-being of Hispanic women, with an intentional focus on serving economically disadvantaged individuals and communities. With its full version released in 2023, SuperVive provides free synchronous and asynchronous content including podcast episodes, wellness videos, recipes, live virtual classes, expert capsule sessions, downloadable guides, guided meditations, personal and group health challenges, forums and other sources of community support. The interprofessional collaborators include integrative medicine clinicians, health coaches, educators, psychologists, chefs, financial experts, nutritionists, personal trainers, and the members of the virtual community. The live classes and podcasts frequently incorporate modalities from diverse traditions including forest bathing, qi gong, yoga and breathwork, spirituality, and herbalism.

### SV Recruitment

The SuperVive membership and most of the application content is available at no cost to all registered participants (a business model known as a freemium). A premium version launched in 2023, where members have access to additional resources like asynchronous classes with experts, group coaching, a meditation center, and a virtual gym for $3.99 per month.

### SV Empowerment Classes

The virtual class series in patient empowerment was implemented in March of 2023 and continues to meet monthly via Zoom. The participants join the live class using a free link provided in the app. Most classes consist of 30 min of didactic content followed by group discussion and experiential learning. The empowerment class series framework encompasses concepts in integrative health, mind-body medicine and positive psychology including opportunities for reflection and collaborative learning.

At the core of the empowerment class series is the study and application of the language of emotions for better connection, both at the individual and collective level. The integrative health equity content includes accessible information on sustainable lifestyles including the intentional practice of rest and relaxation, learning from other traditions, practicing reciprocity, and caring for the planet. Our members are encouraged to tap into their intuition and self-awareness to seek healing partnerships that align with their unique needs and goals - environments where they feel they belong. A list and description of twelve empowerment classes developed by the author for SuperVive-Comunidad can be found in Appendix 1.

## TRW and SV Outcomes Measurements

All participants complete a sociodemographic assessment before starting TRW and/or joining the SV virtual community. The assessment is administered via Google Forms and includes email address, year and country of birth, level of education, civil and employment status, family income, and an optional question on immigration status.

Before starting and after graduating from TRW, the participants complete a Likert-scale survey administered via Google Forms. In the pre and post surveys, the participants self-report their knowledge and performance around healthy eating, mental health, physical activity, empowered health and social connection. The impact of the program in maintaining or enhancing the likelihood to engage in healthy behaviors is calculated by comparing the results before and after the intervention. The post surveys also provide a comments section where participants are encouraged to share their experience with the program.

Similarly, SV members complete a Likert-scale survey at enrollment. A follow up survey for SV participants is administered twice yearly. Members who have joined for 3 or more months are invited to participate. In addition to the questions around healthy behaviors, SV members evaluate the application’s ability to provide culturally relevant and accessible content as part of a wellness-oriented virtual community.

All SV members are invited to complete the surveys independent of the level of participation. An individual account is disenrolled at the member’s request or when a member has not engaged with the application for a period of time exceeding 90 days.

### Program Evaluation Results

As of December 2024, The Rojo Way had served over 1800 individuals and SuperVive had 926 members. Combined, TRW and SV had 1093 direct participants and provided 22 417 hours of wellness education in 2024. In average, each participant reports directly impacting the lives of 5 or more other people.

Ninety-nine percent of the participants identify as female. Ninety-five percent of the members reside in the US, with 89% living in Texas. Five percent of the members live in Latin America including Mexico and Colombia. Eighty-one percent of the participants are between 30 and 59 years old, 80% report being married or living with a partner, and 83% report a household income of less than $50,000 yearly. Seventy-one percent of the participants have not received post-secondary education with 72% reporting no consistent source of income.

Ninety-nine percent of SuperVive members recommend the program, with 95% reporting accessibility and cultural relevance of the content. When combining data from TRW and SV for 2024, 94% of participants report having maintained or improved their physical activity, healthy food choices, and mental health. Ninety percent report feeling part of a positive health community, while 96% of the participants report maintaining or improving their health empowerment.

The SuperVive annual report for 2024 is available online^24^ and shared as a supplemental file.

### Discussion

Hispanics living in the US face challenges in accessing health information and services due to language barriers, cultural differences, social isolation, and economic disadvantages.^10^ These issues contribute to disproportionate rates of cardiometabolic disease, cancer, and poor health outcomes among Hispanics.^10^

Rosa es Rojo’s mission is to create Positive Health Communities where culturally tailored and accessible chronic disease prevention programs can help break down barriers and promote healthier lifestyles. The 2 signature wellness programs, TRW and SuperVive, provide culturally relevant health education and connect Hispanic women to a community focused on individual and collective health, supported by digital engagement. The programs seek to reduce income, language, and education barriers to health and well-being for Hispanic women and their families in the US.

SuperVive Comunidad is primarily serving Spanish-speaking Hispanic women in North Texas. In 2024, most participants reported maintaining or improving healthy eating habits, mental health, and physical activity. These outcomes highlight SuperVive’s ability to support the health and wellbeing of Hispanic women living in the US.

SuperVive’s wellness content is culturally relevant and accessible to participants. Using social connection as a foundational pillar, we aim to scale our digital health platform to improve access to integrative health education and positive outcomes.

The following limitations warrant consideration:

(1) The voluntary nature of participation and self-reported outcomes may introduce selection bias.
(2) The relative lack of unrestricted funding has affected the ability to provide competitive salaries and benefits and limits the resources available to engage in data and outcomes analysis.

Future goals include conducting longitudinal studies of health outcomes and expanding the evidence base for virtual community-based integrative health interventions in Hispanic women. If reinterpreted for other languages and cultures, our model could help advance integrative health equity for other populations. Additional opportunities for geographic and digital expansion include employee wellness programs for Hispanic women as well as partnerships with health insurance companies, academic institutions, and employers.

In 2025, Rosa es Rojo rebranded as SuperVive, to better reflect the organization’s mission to empower the health and wellbeing of Hispanic individuals and communities. SuperVive is collaborating with various employers, safety net hospitals and other institutions to advance the learning and practice of integrative health equity and its impact in accessing health, enhancing workplace wellbeing, and achieving positive outcomes.

### Conclusion

The success of SuperVive Comunidad demonstrates the potential of culturally tailored, technology-enabled health education programs to address health care disparities and advance integrative health equity in the Hispanic community. The high engagement rates and positive outcomes suggest that combining accessible digital platforms with culturally relevant content can help to reduce traditional barriers to health education.

## Supplemental Material

Supplemental Material - SuperVive-Comunidad App: Advancing Integrative Health Equity for the Hispanic Community Through Social Connection Supplemental Material for SuperVive-Comunidad App: Advancing Integrative Health Equity for the Hispanic Community Through Social Connection by Arlene Betancourt in Global Advances in Integrative Medicine and Health

## Appendix 1

The following empowerment classes were presented live from March of 2023 through April of 2024. The recorded videos are available in the SuperVive app.

*La intuición y tu salud – Intuition and your health* was originally presented as a podcast and became the first empowerment class. The participants are introduced to the concept of interoception, its relationship to complex health and disease processes, and how to listen to our bodily signals while cultivating awareness.

*Superviviendo la vergüenza con autocompasión y empatía* - *Overcoming shame with self-compassion and empathy* discusses the differences between guilt and shame and how chronic shame might lead to health care avoidance. The participants are introduced to tools that support the development of shame resilience including the practice of compassion and mindful self-compassion.

*Cómo seleccionar tu médico primario – How to select your primary care clinician* explores the different health professionals that provide primary care and how to choose a primary care team that aligns with the individual’s unique needs and goals of care.

*Integrando la medicina integrativa y la medicina convencional –* Inspired by the National Center for Complementary and Integrative Health, this class explores the differences and similarities between alternative, complementary and integrative medicine including a case-based learning activity that showcases accessible integrative modalities for the treatment of hypertension.

*Rituales de conexión para una salud holística - Rituals for connection and holistic health* introduces the participants to the concepts of centering, grounding and reciprocity. Accessible ways of creating a personalized self-care routine are explored, such as engaging in ceremonies and other healing circles, reframing limiting narratives, and practicing reciprocity.

*SuperVive con estilos de vida saludables y sostenibles – SuperVive with healthy and sustainable lifestyles* introduces the 6 pillars of lifestyle medicine including ideas for implementation. The class ends with a virtual forest bathing experience, highlighting the importance of social connection and contact with nature for optimal health.

*Creando conciencia sobre el cáncer - Creating awareness about cancer* highlights modalities available for breast, cervical, and colon cancer screening and lifestyle behaviors that help reduce the risk of cancer and other chronic diseases.

*Disruptores endocrinos: qué son y cómo evitarlos – Avoiding endocrine disruptors* explores the ubiquitous presence of this nocive chemicals and their association with multiple health conditions. The class provides accessible ideas and resources to help minimize exposure to toxicants.

*Superviviendo con el mindfulness: Buenas Días, Te Quiero –* In our first book club, we discuss the book *Good Morning, I Love You* authored by Shauna Shapiro, PhD. The class introduces the core elements of mindfulness with a focus on curiosity and self-compassion.

*Empoderados con la psicología positiva –* This introduction to the field of positive psychology showcases the core elements of wellbeing as defined by Martin Seligman, PhD. The participants are introduced to the intentional practice of positive emotions and the benefits of cultivating positive relationships and social connection.

*Empoderados con la lectura: El Atlas del Corazón -* This class dives deeper into the world of emotions, as defined by Brené Brown, PhD, LMSW in her book *Atlas of the Heart*. Based on the feedback received during previous classes, this session contrasts social isolation vs positive solitude and provides tools for the participants to help adolescents to better understand their emotions. The lesson shares practical steps to establish healthy boundaries that align with the individual’s values and purpose.

*SuperVive con el sueño y el descanso - SuperVive with better sleep and rest* discusses the importance of restorative sleep for optimal health and explores the challenges many modern workers experience including chronic stress, burnout, and workism. The class provides an accessible plan to customize a personalized sleep routine including when to consult a clinician.
