# Supplementary material for: SuperVive-Comunidad App: Advancing Integrative Health Equity for the Hispanic Community Through Social Connection
Source: Glob Adv Integr Med Health. 2025 May 23;14:27536130251345428. doi: 10.1177/27536130251345428 (PMC12103669; doi:10.1177/27536130251345428)
Supplement: Supplemental Material - SuperVive-Comunidad App: Advancing Integrative Health Equity for the Hispanic Community Through Social Connection [file sj-pdf-1-gam-10.1177_27536130251345428.pdf]

SUPERVIVE

IMPACT REPORT

2024

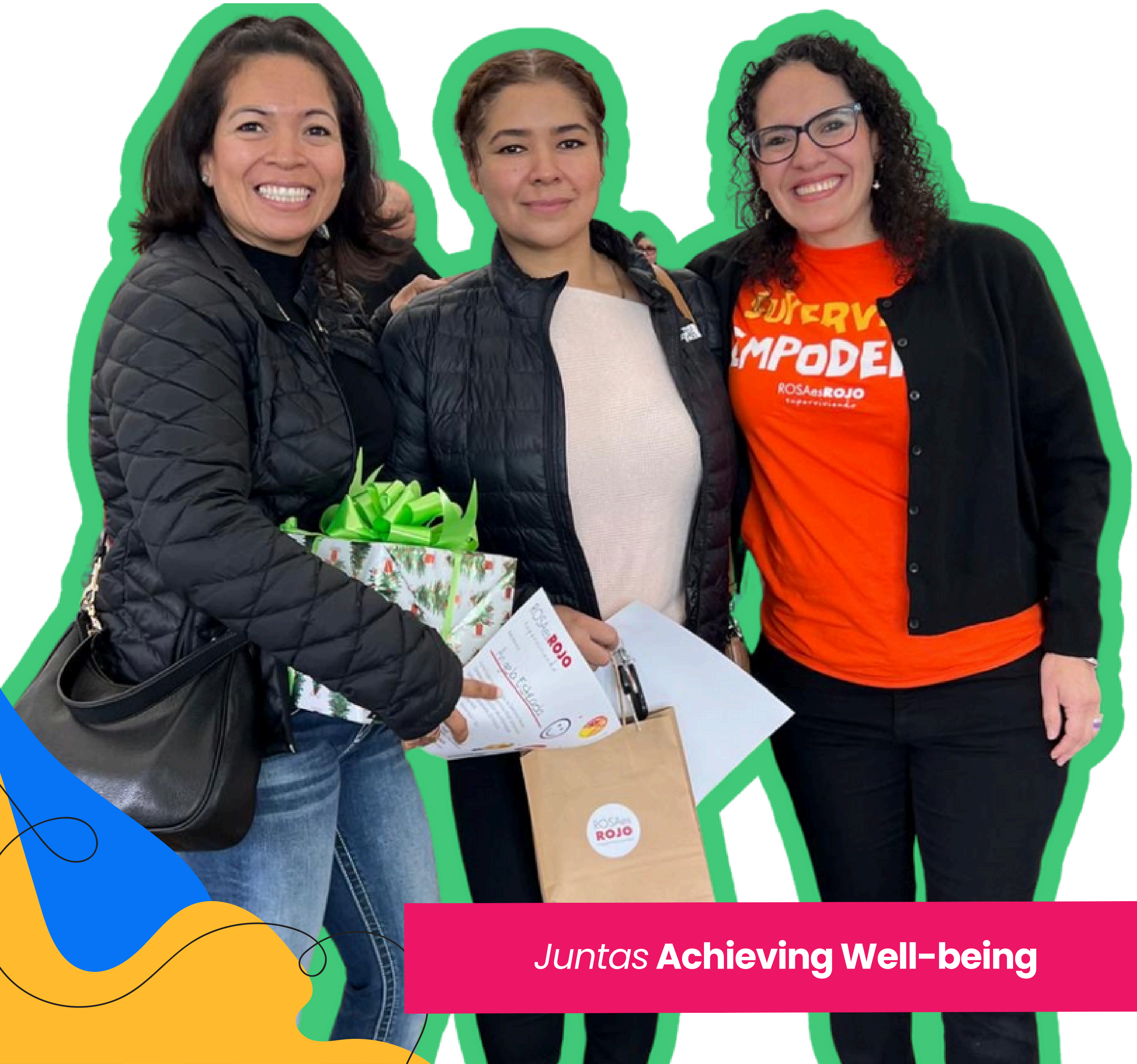

Juntas **Achieving Well-being**

A REFLECTION FROM AIDEÉ

Dear Friends,

2024 has been remarkable! Our wellness programs have grown stronger, allowing us to better serve the Hispanic women we support—women who are transforming their lifestyles, overcoming healthcare barriers, embracing healthier lives, and fostering collective well-being. This progress has been possible because of you! In this Impact Report, you'll find inspiring stories and key outcomes that prove we are on the right path.

A New Chapter: The Evolution of ROSAesROJO

Since 2016, we've empowered Hispanic women and their families to take control of their health, prioritizing prevention over intervention. Originally focused on cancer prevention, our mission expanded three years ago to include chronic disease prevention, broadening our reach and impact.

Today, I'm thrilled to announce our transition from ROSAesROJO to SuperVive—a name that better reflects our mission and growing impact. My personal journey—as a cancer survivor and immigrant navigating the challenges of the healthcare system—remains foundational. But today, it stands alongside our participants' powerful stories, and you will love this!

Introducing SuperVive: A Name That Represents Us All

SuperVive, already recognized through our SuperVive Comunidad App and SuperVive Podcast, embodies the voices of the 2,800+ women who have built this thriving wellness community—and the many more to come.

While our mission remains unchanged, this rebranding strengthens how we represent our work today and our vision for the future.

Our refreshed logo, with its vibrant colors and bold "V", symbolizes empowerment, unity, and inclusivity—because we are "***Juntas, achieving well-being.***"

Scaling Our Impact Together

We are beyond excited! This transition marks a new chapter in scaling our community-based, peer-support health education model—one that teaches wellness habits, prevents chronic diseases, and dismantles health inequities.

Healthcare Is a Human Right

We believe healthcare is a human right, not a privilege reserved for a few. Every individual deserves access to quality healthcare and education, regardless of background, immigration status, or socioeconomic level. And SuperVive is leading the way, making this vision a reality—more powerfully than ever before.

Let's build a community that heals and transforms lives—together.
Thank you for being part of this journey.

With gratitude,
Aideé

SUPERVIVE

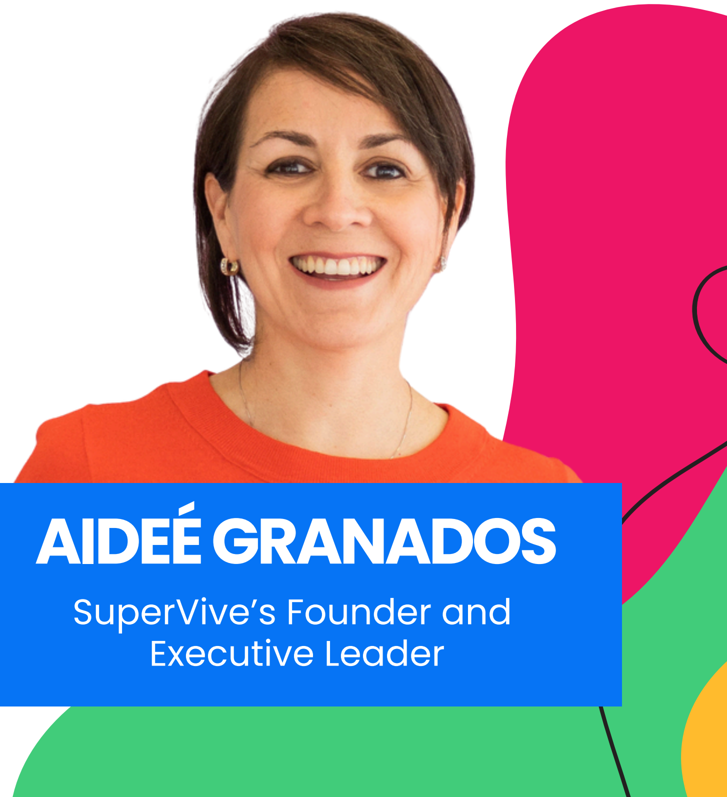

AIDEÉ GRANADOS
SuperVive's Founder and Executive Leader

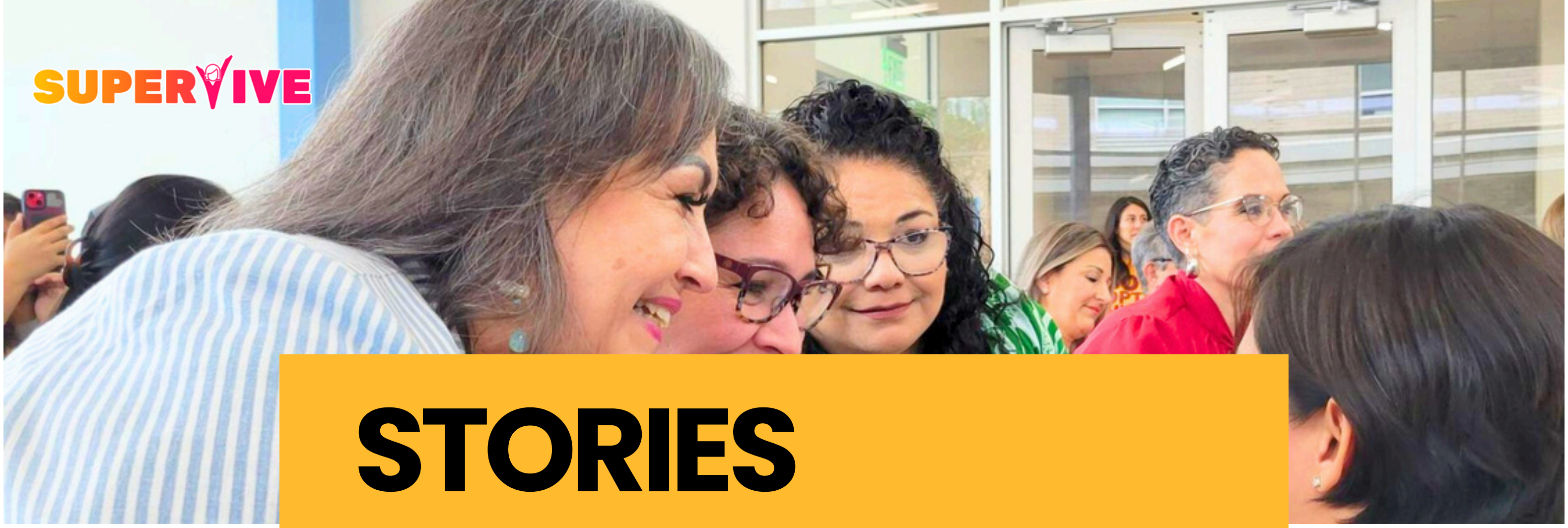

STORIES THAT *EMPOWER*

Get to know Angélica

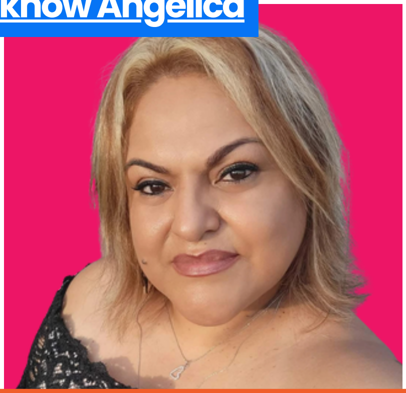

Angélica's Transformation: Health Empowerment

Get to know Silvana

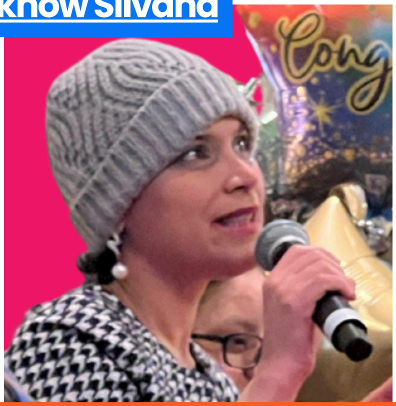

Silvana's Transformation: Empowerment and Hope

Get to know Aracelis

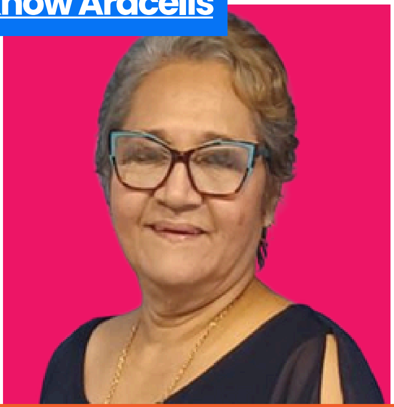

SuperVive Podcast: From the Garden to the Plate with **Aracelis**

Get to know Rocío

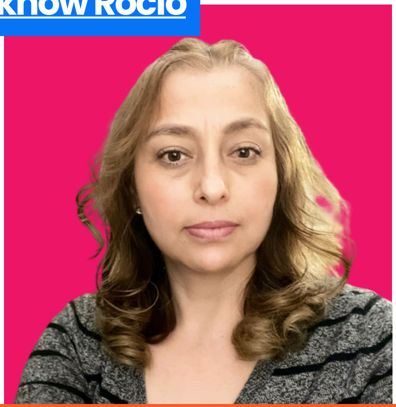

SuperVive Podcast: Eating Delicious, Simple, and Nutritious Food with **Rocío**

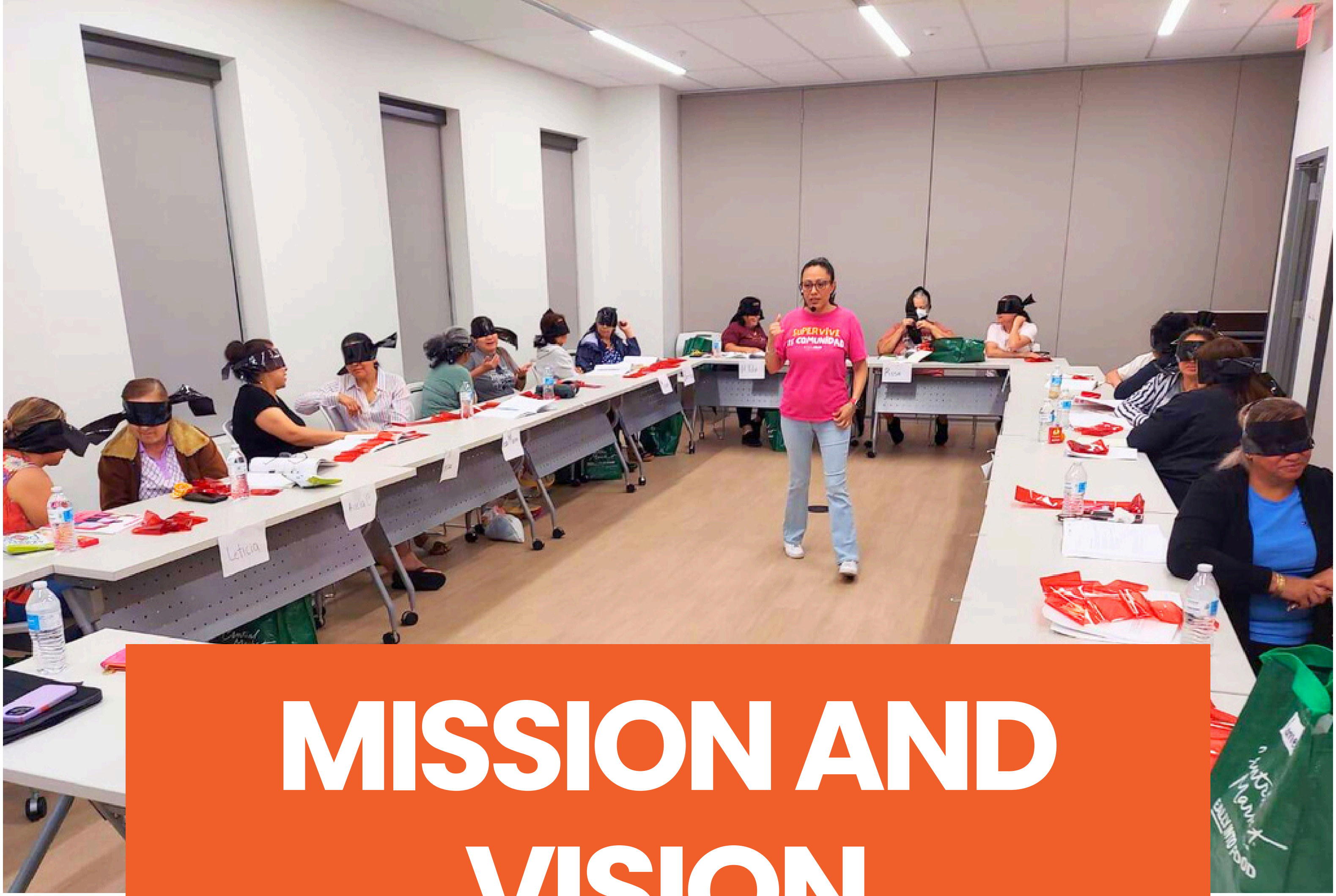

MISSION AND VISION

MISSION

SuperVive provides health and well-being education to Hispanic women and their families in the United States by **creating Positive Health Communities** where culturally tailored and accessible chronic disease prevention programs are at the center.

We use four pillars to drive equitable whole health behavioral change: **Nutrition, Mental Health, Physical Activity and Empowered Health.**

VISION

- **SuperVive** dismantles income, language, and education barriers to health and well-being for Hispanic women and their families in the U.S.
- We are leading Hispanics to **live healthier lives** and reduce the incidence of chronic diseases among them.

SOCIODEMOGRAPHY

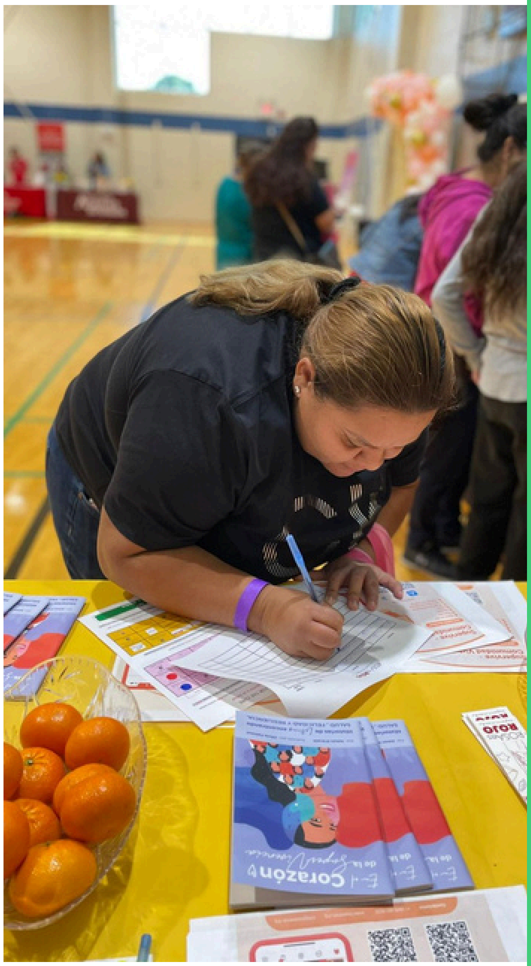

99% are women

1% are men

81% are between 30 and 59 years old

80% are married or live with a partner

83% have a household income of less than $50k dollars a year

72% do not work or do not have a permanent individual income

71% have a high school level of education or less

In 2024:

89% of our program participants **live in Texas**

6% of the participants **live in other states of the United States** (NY, CA, VA, mainly)

5% of the participants (especially in SuperVive Comunidad) live in other countries such as **Mexico, Colombia, Canada, Spain among others.**

In Texas:

67% Dallas County

12% Tarrant County

6% Collin County

5% Denton County

10% Other Counties

SUPERVIVE

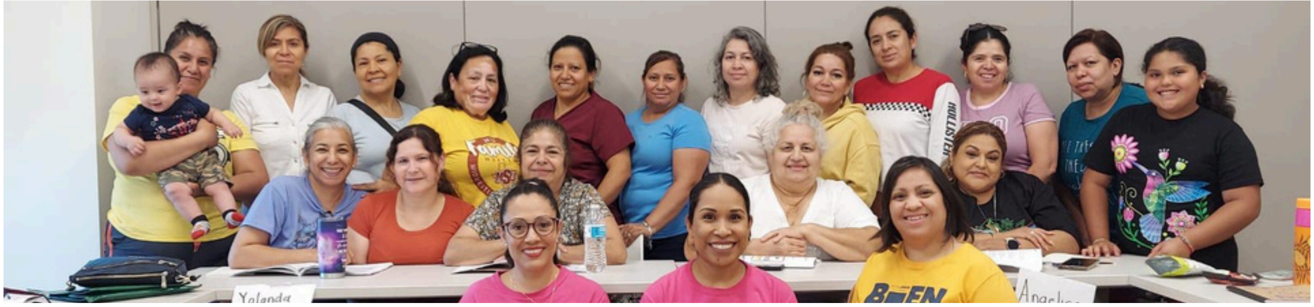

SOCIAL DETERMINANTS OF HEALTH

We have identified four main barriers to accessing health and well-being in the Hispanic community:

1 LANGUAGE

* **81% of Hispanic adults** in the United States prefer health professionals who speak Spanish.
* **Only 7% of medical doctors in the U.S.** and 9% of health professionals are Hispanic.
* **Sometimes Hispanics rely on their children as interpreters.**
* **Almost 50% of Hispanics say they are unhealthy because they do not understand health instructions** due to cultural and language differences. (PRC, 2022)

2 COMMUNITY-BASED NETWORKS

* **Numerous Hispanic immigrants confront obstacles and threats beyond their control.**
* **Hispanic immigrants,** particularly women, **experience greater social isolation and loneliness** than U.S.-born Hispanics and other groups, resulting in increased rates of adverse health outcomes. (CDC, 2021)
* **Loneliness increases the chances of premature death** by 20%: about the same effect as obesity. (The Guardian, 2016)

3 INCOME

* **25% of Hispanics in the United States** live below the poverty line. (KFF, 2021)
* **20% do not have health insurance;** More than double that of their white peers (KFF, 2021)
* **Legal status can be a barrier** to higher income and financial stability. (CMS, 2019)

4 CULTURE

* **Fear and shame are emerging** as social determinants of health, impacting access to care and health outcomes.
* **Fear often stems** from factors like immigration status, language barriers, and unfamiliarity with healthcare systems.
* **Shame arises from cultural stigmas** and taboos surrounding medical check-ups and health conversations. (NLM, 2017)

SUPERVIVE

SUPERVIVE

HEALTH CONSEQUENCES

These barriers are causing:

Cancer accounts for the
main cause
of mortality and morbidity
in Hispanics in the U.S. (ACS, 2022)

70% of Hispanic
adults in the U.S. are more likely to be **diagnosed with diabetes.** (OMH, 2018)

60% of people
in the U.S. have at least **one chronic illness.** (CDC, 2023)

46% of Hispanic
adults in the U.S. are **affected by obesity.** (CDC, 2021)

39% of Hispanic
adults in the U.S. live with **high blood pressure.** (CDC, 2021)

23% of Hispanics
experienced **suicidal thoughts.** (CDC, 2021)

BUT GUESS THE GOOD NEWS...

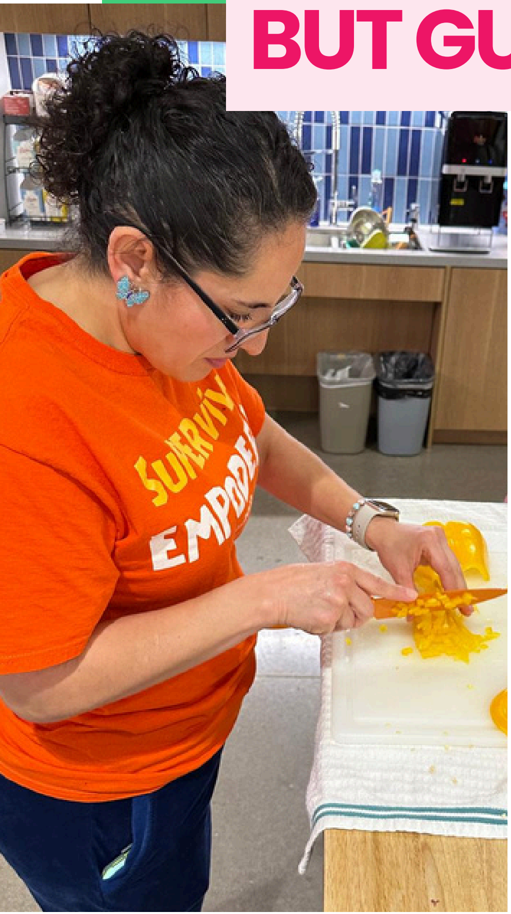

Lifestyle choices and ***comprehensive health behavior*** changes represent **34%** of the person's health. **(Hood, et al 2016)**

The other 47% depends on the social determinants of health, and the rest is determined by clinical treatments and the environment.

THERE IS POWER IN OUR HANDS!

SUPERVIVE

| *Programs*

SUPERVIVE COMUNIDAD

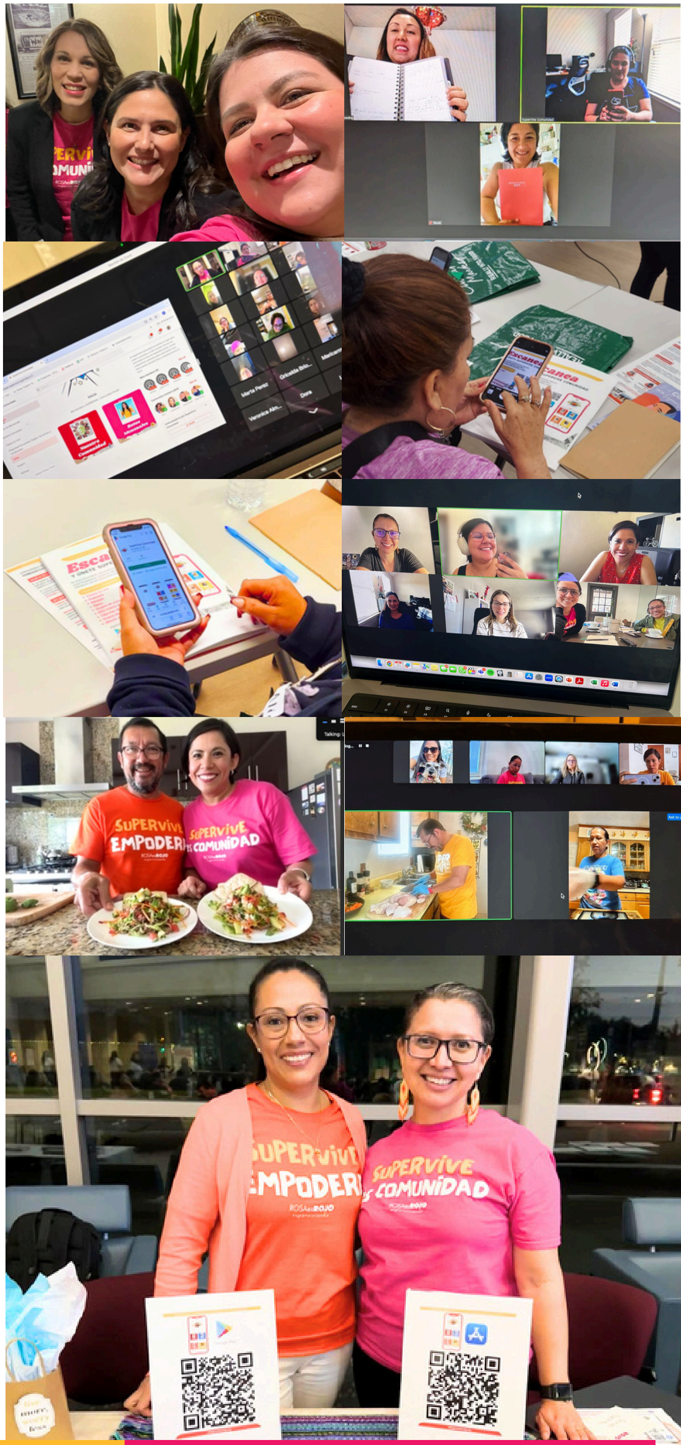

SuperVive Comunidad is an app in Spanish with both asynchronous and synchronous content, building a virtual community to **empower the health and well-being of Hispanic women.**

SuperVive Comunidad emphasizes taking responsibility for personal and community health. **Through this program, we leverage technology for community benefit,** creating networks of support and responsibility and **connecting women in a community of well-being to reduce chronic diseases.**

WHAT YOU CAN FIND:

- Podcast
- Live Classes
- Expert Capsules
- Support Groups
- Wellness Videos
- Recipes
- Healthy Challenges
- Book Club
- Meditations
- Virtual Gym

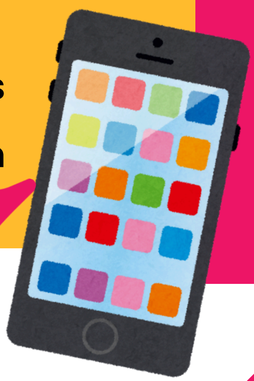

Find a GREAT community to share, inspire and get closer to health!

Available for FREE:

SUPERVIVE

| *Programs*

THE ROJO WAY

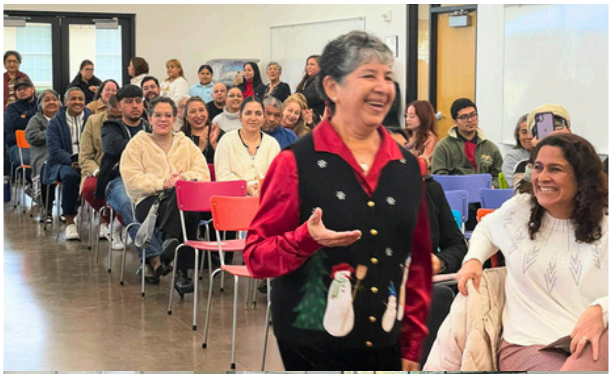

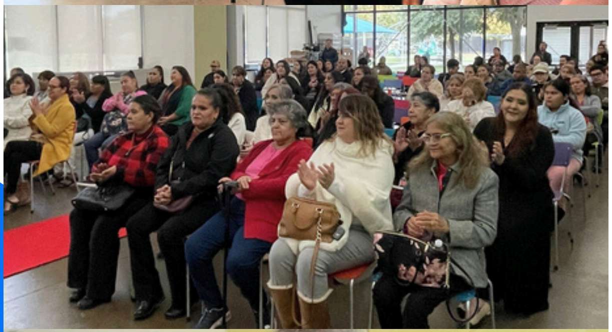

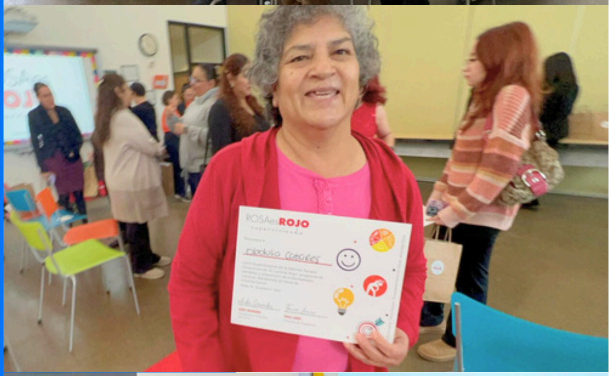

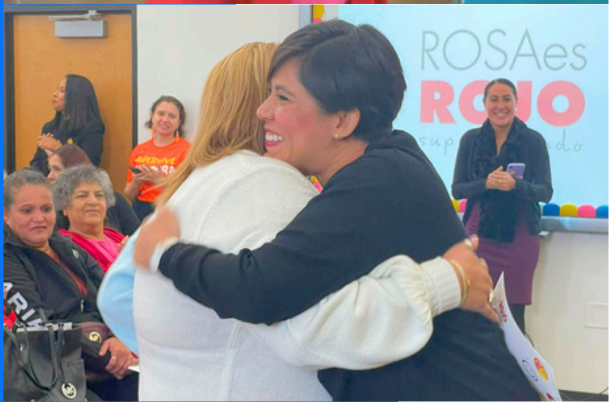

HEALTH EDUCATION FOR HISPANIC WOMEN

The Rojo Way offers direct health and wellness education designed to prevent chronic diseases, either in person or online.

100%
in Spanish

100%
Culturally Relevant

This 23-hour program is comprised of **interactive workshops designed using the Accelerated Learning Methodology**, and of individual mentoring sessions where **our Ambassadors act as accountability partners** to help participants develop a personalized action plan, goals, and reflect on progress.

19 hours
of interactive workshops

4 hours
of individual mentoring sessions

OUR PROGRAMS IMPACT

Despite facing numerous health challenges, Hispanics in the United States **tend to have a longer life expectancy and lower mortality rates** compared to other groups. This is especially noticeable among less acculturated immigrants, highlighting how traditional lifestyles from our home countries contribute to better health outcomes.

Acculturation has both benefits and drawbacks. On the downside, we often adopt a Western diet, which is typically less healthy, and may lead more sedentary lifestyles. Additionally, **mental health remains a taboo topic in many Hispanic communities**, making it harder for people to seek the help they need. While adapting to a new culture is natural and can bring positive changes, maintaining our cultural traditions—alongside access to relevant wellness resources—**helps build a healthier community.**

Encouraging healthy habits and providing culturally relevant content in Spanish are essential steps in supporting the well-being of Hispanics in the U.S.

*Sources: * NCBI, 2021* Novant Health, 2020*

WELLNESS EDUCATION HOURS

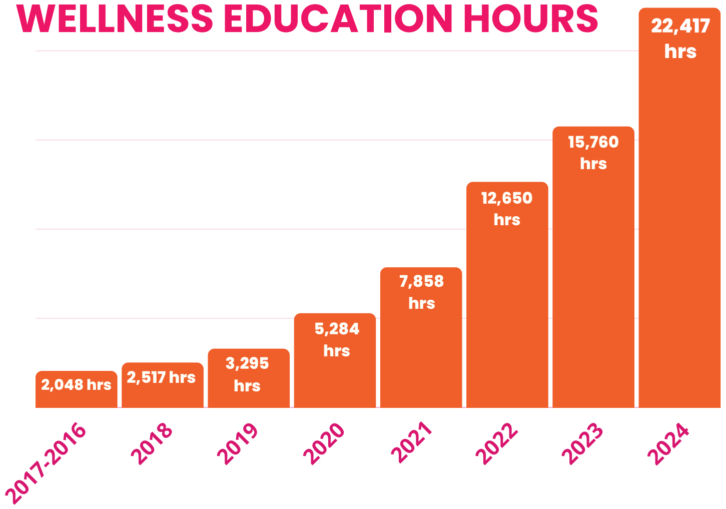

As of December 2024, we have accumulated an equivalent of **more than 71,000 hours of wellness education**

Our programs continue to make a meaningful impact, with the majority of participants maintaining or **improving key aspects of their health and well-being:**

- **94%** improved or sustained **healthy eating habits.**
- **94%** maintained or increased **their physical activity levels**
- **94%** reported **positive mental health outcomes.**
- **96% felt** empowered to take **charge of their health.**
- **91%** experienced overall **improvements in well-being.**
- **90%** felt a strong sense of connection and support **within the wellness community**
- **95%** found the **wellness resources culturally relevant.**
- **95%** said our **programas were easy to use and accessible.**

99% of our participants RECOMMEND our programs

Total direct participants in 2024:

1,093

The Rojo Way: **301**

SuperVive Comunidad: **926**

SUPERVIVE

OUR NUMBERS IN 2024

INCOME SOURCES:

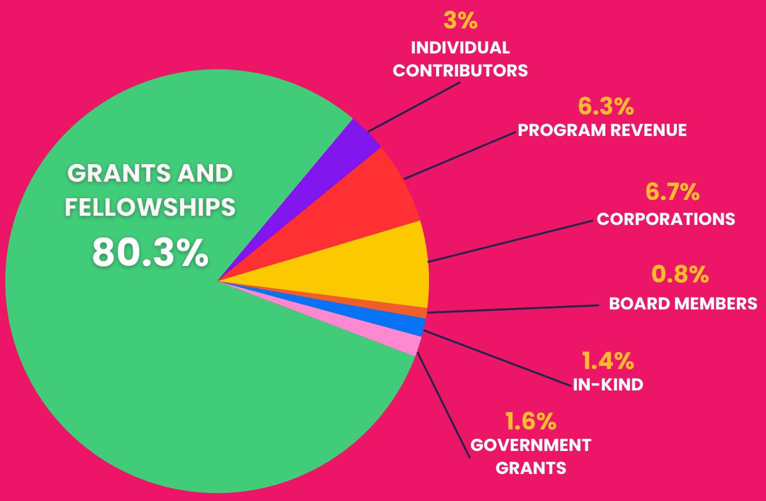

WE INVESTED OUR INCOME AS FOLLOWS:

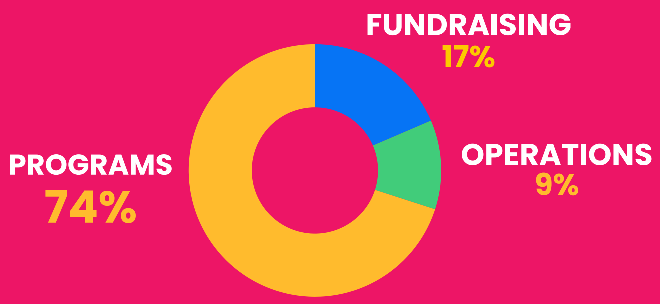

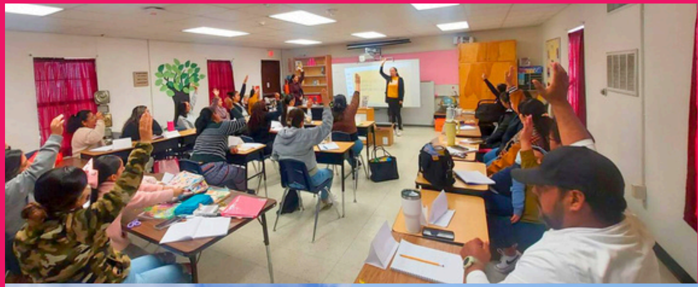

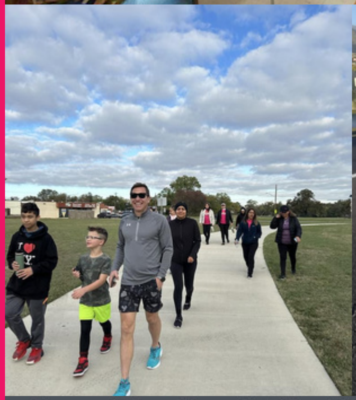

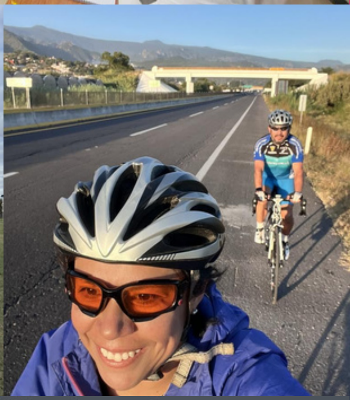

SuperVive Fun Rally: Miles for a Cause

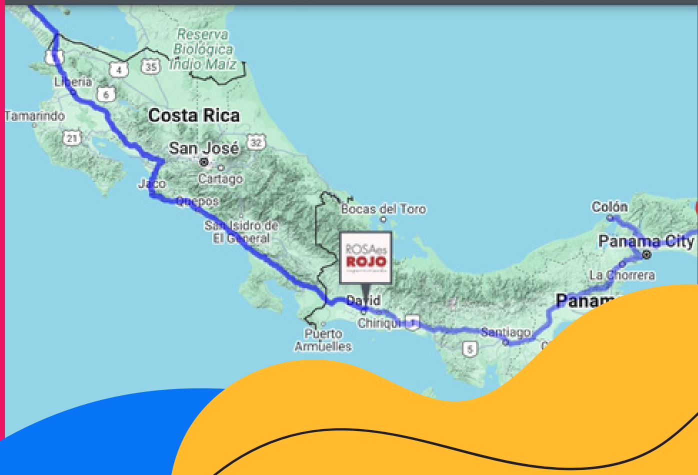

TOTAL INCOME OVER THE YEARS (FORM 990)

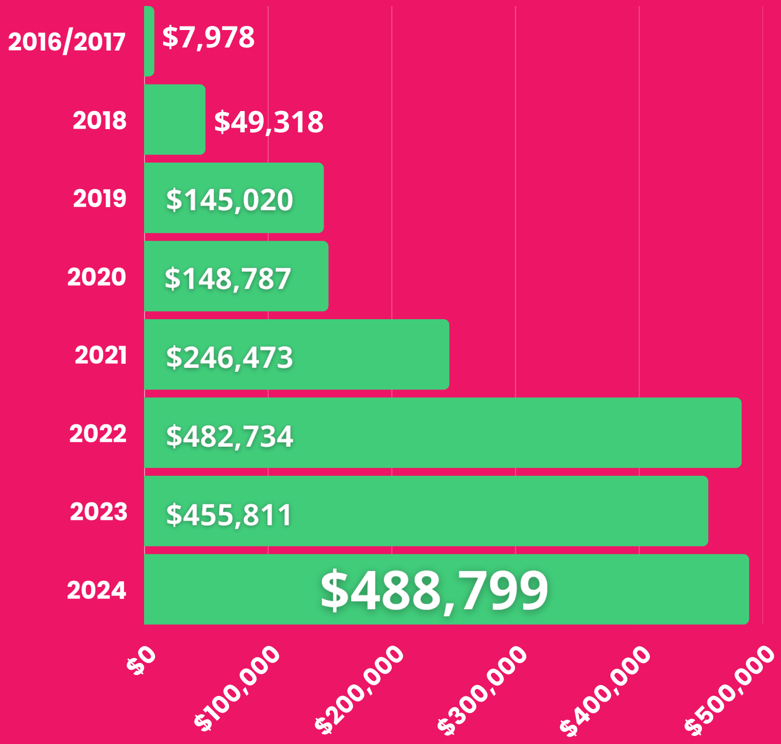

- Amounts expressed in U.S. dollars.
- Starting in 2021, the accounting system changed from “Cash” to “Accrual”.
- The 2024 data may vary after the final review of the Independent Auditor.

Big News!

SUPERVIVE CANNENTA foundation **We were selected for Texas Health's Community Impact Grant!**

We're honored to be chosen for the Texas Health Community Impact grant, a significant investment in North Texas! Through this support, SuperVive, in collaboration with the **Cannenta Foundation**, will expand culturally tailored mental health services for Hispanic women in Dallas.

This grant enables us to enhance our wellness app, connecting underserved women with affordable, accessible mental health resources, counseling, and essential services. Together, we're breaking barriers to health equity!

Texas Health Community Hope

OUR 2025 PLAN

Our 2025 plan **focuses on strengthening Supervive Comunidad App, expanding its reach in the region**, and surpassing 1,200 members. **We are also expanding our faculty team, bringing in wellness experts from across the U.S. and other countries.**

We are forging **strategic partnerships with corporate partners** to introduce Supervive Comunidad App into workplaces, showcasing how well-being and productivity go hand in hand. This initiative, designed for workplaces with a predominantly Hispanic female workforce, is called **"Thriving Teams."** Additionally, we are collaborating with Hispanic ERGs from major corporations to deliver culturally relevant health and wellness programs.

Exciting innovation projects are coming to Supervive Comunidad App, **leveraging technology to enhance health and well-being.** We will train our ambassadors in mentorship skills and launch a live chat support line, ensuring that the community experience is real, supportive, and accessible to more people.

The Rojo Way will also continue in 2025, with a stronger regional focus and in-person cohorts in collaboration with our community partners, aiming to serve over 200 new women.

Stay tuned for the return of the Supervive Rally 2025, an expanded social media presence, and more empowering stories from our participants on our podcast. We will continue working hand in hand with our board of directors, staff, volunteers, ambassadors, corporate partners, donors, and foundations to **keep making a difference.**

SPECIAL THANKS

TO OUR DONORS AND COMMUNITY PARTNERS

TO OUR BOARD OF DIRECTORS

- Jenny Apperti, Board Chair
- Barbara Arcobelli, *Secretary*
- Adriana Mendez-Rugh, *Treasurer*
- Adela Santana
- Arlene Betancourt
- Betty Cardiel
- Blanca Montellano
- Citlali Sarabia
- Diana Charbonneau
- Gildardo Zafra
- Josue Mendez
- Zach Garrison

TO OUR PARTICIPANTS, VOLUNTEERS, AMBASSADORS, STAFF AND TO YOU, FOR SUPPORTING US AND READING US.

IF YOU WANT TO SUPPORT SUPERVIVE

DONATE HERE

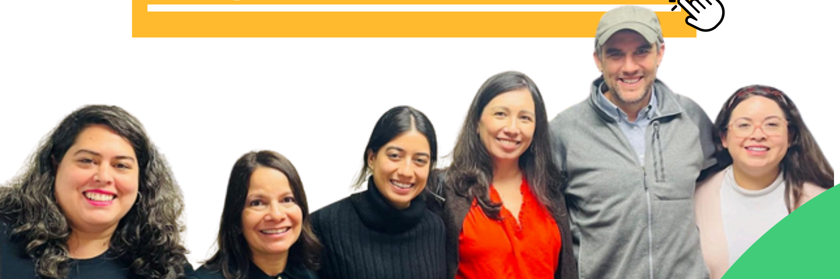

CONTACT

supervive.org

info@rsupervive.org

@superviveorg

P.O. Box 250435

Plano, TX 75025-0435

+1 (469) 431-0232
